# A theoretical alternative intraosseous infusion site in severely hypovolemic children

**DOI:** 10.4102/phcfm.v7i1.835

**Published:** 2015-07-23

**Authors:** Nkhensani Mogale, Albert-Neels van Schoor, Marius C. Bosman

**Affiliations:** 1 Department of Anatomy, Sefako Makgatho Health Sciences University, South Africa; 2 Department of Anatomy, University of Pretoria, South Africa

## Abstract

**Background:**

Studies have shown that the venous system tends to collapse during hypovolemic shock. The use of the bone marrow space for infusions is an effective alternative, with the tibial insertion site being the norm.

**Objectives:**

This study was conducted to determine a quick intraosseous infusion method that could be an alternative to the tibial route in neonates during emergency situations.

**Method:**

A sample of 30 neonatal cadavers was dissected to explore a possible alternative to the tibial insertion site. The needle was inserted in the superolateral aspect of the humerus. The needle infusion site was then dissected to determine possible muscular and neurovascular damage that might occur during the administration of this procedure, with the greatest concern being the posterior circumflex humeral artery and axillary nerve exiting the quadrangular space. The distance of the needle insertion site was measured in relation to the soft tissue as well as to bony landmarks.

**Results:**

The calculated 95% confidence interval shows that the needle can be safely inserted into the intraosseous tissue at the greater tubercle of the humerus 9.5 mm – 11.1 mm from the acromion. This is about a little finger’s width from the acromioclavicular joint.

**Conclusion:**

Anatomically, the described site is suggested to offer a safe alternative access point for emergency infusion in severely hypovolemic newborns and infants, without the risk of damage to any anatomical structures.

## Abstract (fr)

**Point de perfusion intra-osseux alternatif théorique chez les enfants gravement hypovolémiques**.

**Contexte:**

Des études ont montré que le système veineux tendait à l’effondrement lors d’un choc hypovolémique. L’utilisation de l’espace de la moelle osseuse pour des perfusions est une alternative efficace, le point d’insertion tibial étant la norme.

**Objectifs:**

Cette étude a été menée afin d’établir une méthode de perfusion intra-osseuse rapide qui pourrait être une alternative à la voie tibiale chez les nouveau-nés lors de situations d’urgence.

**Méthode:**

Un échantillon de 30 cadavres néonatals a été disséqué pour explorer une alternative possible au point d’insertion tibial. L’aiguille a été insérée dans la partie supéro-externe de l’humérus. Le point de perfusion de l’aiguille a ensuite été disséqué afin de déterminer d’éventuels dommages musculaires et neurovasculaires qui auraient pu se produire lors de l’administration de cette procédure, la plus grande préoccupation étant l’artère circonflexe humérale postérieure et le nerf axillaire sortant de l’espace quadrangulaire. La distance entre le point d’insertion de l’aiguille a été mesurée par rapport au tissu mou et à des repères osseux.

**Résultats:**

L’intervalle de confiance à 95% calculé montre que l’aiguille peut être insérée en toute sécurité dans le tissu intra-osseux au niveau du tubercule majeur de l’humérus à 9.5 mm – 11.1 mm de l’acromion. Ceci représente environ la largeur d’un petit doigt à partir de l’articulation acromio-claviculaire.

**Conclusion:**

Sur le plan anatomique, on suggère le point décrit afin d’offrir un point d’accès alternatif sûr pour la perfusion d’urgence chez les nouveau-nés et les nourrissons gravement hypovolémiques, sans risque de dommage aux structures anatomiques.

## Introduction

Proper fluid resuscitation is needed in most life-threatening situations and it is of paramount importance that appropriate access is obtained as quickly as possible and without complications. Several studies^1,2,3,4,5,6^ have shown that the venous system tends to collapse during life-threatening conditions, making the normal route of infusion unpredictable and unreliable. Health care workers often work under strenuous conditions and need a safe, quick and reliable method to administer fluid resuscitation. This study was conducted to determine an intraosseous (IO) infusion site different from existing access points as a possible alternative infusion route in severely hypovolemic newborns and infants.

The use of the bone marrow space as an infusion site was initially proposed by Drinker et al. when they described the sternum as a potential site for transfusions.^1,2^ Later, in 1940, Tocantins^3^ described the administration of fluids into the general circulation via the bone marrow using animal experiments to demonstrate this phenomenon. In the same year, Tocantins and O’Neill^4^ documented 14 cases that involved the injection of blood, plasma or saline into the sternal bone marrow of adults and the marrow of the tibia or femur in infants. In 1942, Papper^5^ described access to the marrow space for the use of intravenous (IV) fluids. This led to investigations into the administration of drugs and other fluids with the IO space as an access route to the central circulation. In his study, Papper also stressed the inaccessibility of veins in very young patients and that long periods of continued fluid therapy frequently produce thrombosis of veins with the associated possibility of infection or embolism.^5^ These observations suggested that an alternative route, such as IO infusion, would be of great value.

IO infusion is as quick as with central lines and even faster than with peripheral lines. The use of the IO space for patient resuscitation reached its peak during World War II, when it was commonly used in soldiers experiencing haemorrhagic shock.^7^ Use of IO infusion methods became rare after the war, but the practice was revived in the 1980s and the method has been in use since then. The increased use of this method over the years can be attributed to the evolution of technology that allows for easier penetration of the dense adult bone cortex.^2,7^

The speed of vascular access plays an important role in preventing death and irreversible organ damage. Central lines are often not used in children owing to the difficulty experienced when accessing the venous system compared to in adults, and health care workers need advanced skills to place central lines safely and successfully in children. Primary health care facilities in developing countries are often underequipped to handle the installation of central venous lines, which makes a simple alternative attractive.^8^ In addition, the difficulty with venous access, particularly in a shocked child, necessitates the need for a quick alternative.

IO insertion can be used for administration of fluids to a haemodynamically shocked neonate in whom attempts to access the vascular system have been unsuccessful. Under these conditions, it is usually impossible to administer drugs through peripheral veins, because insufficient perfusion would most probably cause them to collapse.^9^ Rapidly securing vascular access may be a considerable challenge in the presence of shock or intravascular volume depletion, causing peripheral venous shutdown.^10^ This is associated with cardiopulmonary arrest, oedema and extensive burns.^8^ IO insertion may also be useful in older children, as they can become dehydrated very quickly. This is especially relevant in the case of gastrointestinal infections, which occur often in South Africa. External jugular insertion is associated with high malposition rates and is particularly difficult in the very young owing to these patients’ short necks. Several serious complications may also arise, including laceration of the internal jugular vein and infection.^2^ The other preferred route in newborn babies is the umbilical vein, but direct drug injection into this structure has proved to be dangerous.^9^ IO administration is second to that of the umbilical vein, but if the umbilical vein is not available then this route becomes a viable option.

IO vascular access permits the administration of life-saving fluids and medications in critically ill patients. Drugs administered intraosseously enter the circulation as fast and in the same concentration as those administered intravenously.^8^ Current technology allows IO infusion to be considered as a preferential safe and effective route of drug administration in cardiac arrest cases, and should also be considered the first alternative to failed IV access.^2^

IO infusion has proven successful in neonates when the anteromedial surface of the tibia was used as the insertion site.^8^ The technique is simple because the landmarks are easily palpable.^8^ Similar methods have been explored in adults,^10^ with both the proximal humerus (superolateral aspect of the greater tubercle) and the tibia being successfully infused.

It has been shown that in adults, the rate at which the infused contents enter the systemic circulation is comparable whether an IO insertion site is used on the proximal humerus or on the tibia.^7^ The current study is an anatomical exploration to determine whether an easily palpable landmark can be identified on the proximal humerus as a possible alternative IO infusion site to tibial infusion in neonates. To our knowledge, this is the first study to simulate an IO infusion method on the proximal humerus of neonates. This method could prove useful during treatment of traumatic accidents where injury to the lower limbs of the neonate would prevent tibial infusion.

## Research method and design

**FIGURE 1:**
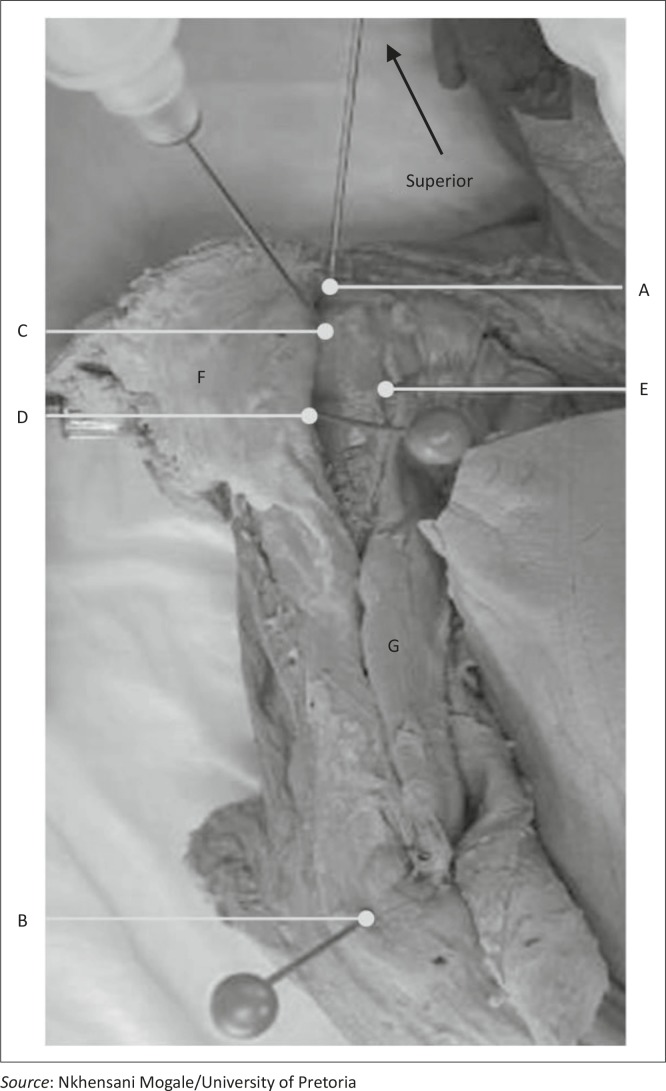
Anterior view of a right neonatal shoulder after the deltoid muscle (F) has been reflected. Pins were inserted into the acromion (A), lateral epicondyle of the humerus (B), the greater tubercle of the humerus (C), the axillary neurovascular bundle as it exits the quadrangular space (D) and the long head of the of the biceps brachii muscle (E). The muscular belly of the biceps brachii muscle (G) is also visible.

**FIGURE 2:**
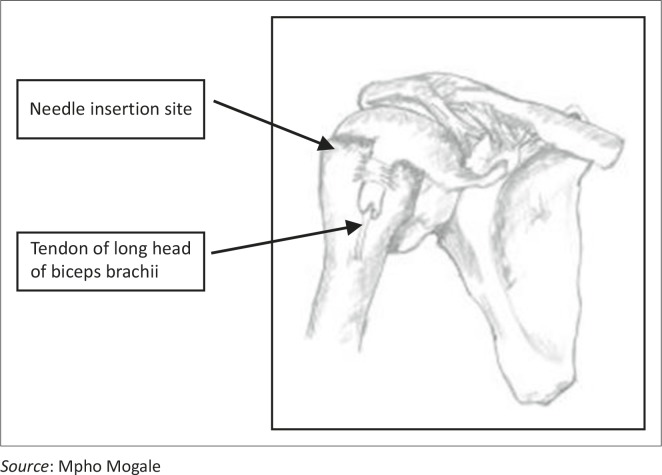
The needle insertion site and the tendon of the long head of biceps brachii muscle shown in the bicipital groove/intertubercular sulcus.

A sample of 30 neonatal cadavers (age range 0–28 days) of low to normal birth weight (1.5 kg or higher) was used in the study. A total of 60 humeri were studied. A 22-gauge Jelco IV spinal needle was inserted at the greater tubercle of the humerus. The area around the insertion site was dissected (Figure 1) to determine the possibility for muscular or neurovascular damage during administration of this procedure.The humerus was chosen because of the large amount of vascular bone marrow that exists in the long bones of neonates and consequently provides access to a non-collapsible venous plexus. (Marrow sinusoids drain into large medullary venous channels, which empty into the systemic venous circulation via nutrient and emissary veins.)^2,7,9^ The needle was inserted in the superolateral aspect of the humerus (as in adults), with the direction of the needle insertion observed and the measured distance between the needle and landmarks recorded (Figure 2). The needle position was then recorded whilst measuring the distance to the soft tissue (neurovascular bundle, long head of the biceps brachii muscle) as well as the relative distances between bony landmarks (acromion and lateral epicondyle of the humerus).

**FIGURE 3:**
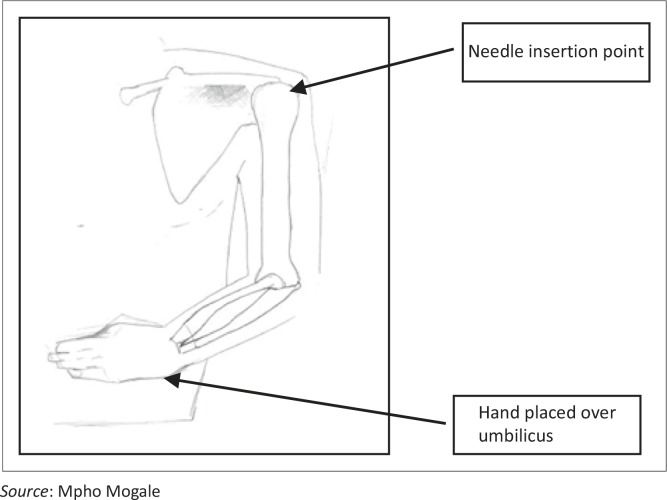
Body in supine position with the humerus adducted against the trunk.

**FIGURE 4:**
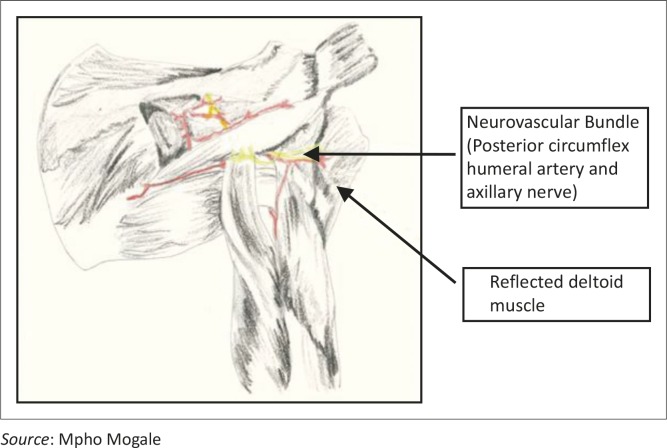
Posterior view of the reflected head of the deltoid muscle with the neurovascular bundle (posterior circumflex humeral artery and the axillary nerve) shown.

Certain standard placement methods were followed. This involved placing the neonatal cadaver in a supine position to present the greater tubercle of the humerus to the investigator. The humerus was adducted against the trunk, with the elbow posteriorly directed towards the table. The hand was secured on the abdomen near the umbilicus (Figure 3). This position provided a more prominent insertion site and ensured protection of the vital neurovascular structures located under the arm. This position also ensures maximal approach to the humeral head.^9,11^ The lateral aspect of the acromion, in line with the acromioclavicular joint, was palpated by feeling the depression when the arm is abducted and then returning to the correct position on the abdomen with the hand near the umbilicus. This position was labelled as point A using a coloured pin. The lateral epicondyle of the humerus (point B) was identified by palpating the distal aspect of humerus and also marked with a coloured pin. The deltoid muscle was then carefully reflected (Figure 4) to expose the neurovascular bundle in the quadrangular space (between the axillary nerve and the posterior circumflex humeral artery) (point D). The long head of the biceps brachii muscle (point E) was exposed within the bicipital groove/intertubercular sulcus. The needle was inserted in the greater tubercle at the mid point of the head (between point A and the surgical neck of the humerus) and labelled as point C.

A sliding calliper of 0.02-mm accuracy was then used to measure the short distances between:

- the acromion (A) and lateral epicondyle (B)
- the acromion (A) and the needle insertion point on the greater tubercle of the humerus (C)
- the needle insertion point on the greater tubercle of the humerus (C) and the lateral epicondyle (B)
- the needle insertion point on the greater tubercle of the humerus (C) and axillary neurovascular bundle (D)
- the needle insertion point on the greater tubercle of the humerus (C) and the long head of the biceps brachii muscle (E).

### Data analysis

A paired *t*-test was used to compare measurements in the left and right humeri. The minimum and maximum distances were recorded and the mean, standard deviation and 95% confidence interval were calculated.

### Ethical considerations

Ethical clearance was obtained from the Research Ethics Committee of the Faculty of Health Sciences, University of Pretoria (reference number S114/2011). As this study made use of human tissue, all the neonatal cadavers were obtained and dissected under the rules and regulations defined in the *South African Health Act* (Act 61 of 2003). All cadavers were donated by the family members or obtained as a result of the deceased being unclaimed by family members. The material was handled with respect and care at all times, and properly safeguarded.

Information regarding the cadaver's age, height and weight was noted and only the researchers conducting the study had access to it. No information that could reveal the identities of the cadavers was obtained.

## Results

A paired *t*-test revealed no significant difference between pairwise measurements of the right and left humeri (*P* > 0.05 for all measurements). Measurements of the right and left sides of the neonatal cadavers were therefore combined to give a sample size of 60 dissected humeri. The results are summarised in Table 1.

**TABLE 1:** Distances of the soft tissue and bony landmarks from the intraosseous needle insertion site on the greater tubercle of the humerus (*n* = 60).

Statistics	A–B (mm)	A–C (mm)	B–C (mm)	C–D (mm)	C–E (mm)
Mean	64.8	10.3	57.4	11.1	8.2
s.d.	9.1	3.1	8.0	2.2	1.7
Range of 95% confidence interval	62.5–67.1	9.5–11.1	55.4–59.4	10.5–11.6	7.8–8.7

s.d., standard deviation.

The mean body length of the cadavers was 0.43 m ± 0.07 m (mean ± SD) and mean weight was 1.69 kg ± 0.74 kg. The calculated confidence interval shows that the ideal insertion point for the IO needle lies 9.5 mm –11.1 mm (mean of 10.3 mm) from A on a line connecting A and B. As the described IO insertion is proposed for application in emergency procedures, the health care worker has to be able to find the site quickly. The insertion point can be determined as the distance equal to the width of the little finger (fifth distal phalanx) from the acromioclavicular joint (approximately 11 mm). The results also suggest that a health care worker should be able to perform the procedure without injury to the important neurovascular structures in that area (posterior circumflex humeral artery and axillary nerve). The results showed that the point of the needle insertion is on average 11 mm from the axillary nerve and accompanying posterior circumflex humeral vessels as they run through the quadrangular space (range: 10.5 mm – 11.6 mm; 95% confidence level), and 8 mm from the long head of the biceps brachii tendon running in the intertubercular groove (range: 7.8 mm –8.7 mm; 95% confidence level).

## Discussion

The vascular network of the long bones provides an ideal route for IO infusion. Drugs or fluids injected into the bone marrow cavity in the proximal humerus pass into the venous sinusoids to the central venous channels and move into the systemic circulation through the nutrient veins. This route is quickly accessible and does not collapse during shock, in contrast to what is often seen in the venous system. Of the drugs tested to date, none has proven unsuccessful for use by IO infusion in neonates.^11,12^ This success can be attributed to neonates having a highly vascular bone marrow in their long bones, which contains a network of non-collapsible venous sinusoids in the medullary cavity of the spongy bone that drain into the venous circulation.^13^ Although technological developments have made this method practically applicable, risks such as disturbance of the proximal humerus growth plate, pain during infusion and potential for infection in the affected bone^13,14^ cannot be eliminated completely. However, these risks have to be weighed against the benefits and the best infusion method should be explored. Therefore, this procedure should be performed once all the risk factors have been considered and it has proved to be the most beneficial method of treatment for the patient.

IO infusion is indicated for neonatal fluid resuscitation in life-threatening situations when attempts at obtaining vascular access elsewhere have been unsuccessful. The IV route of administering drugs and fluids is generally the preferred choice, but can be difficult in obese and oedematous children as well as in children experiencing cardiac arrest or circulatory shock.^8,14^

Our measurements show the upper and lower limits of the distance from C to E to be 8.7 mm and 7.8 mm, respectively. This suggests that the IO needle can be inserted about 5 mm into the bone after initial contact before care must be taken to avoid contact with the epiphyseal growth plate. The epiphyseal growth plate separates the proximal head of the humerus from the shaft and is located distal to the greater tubercle of the humerus. Care must be taken not to angle the needle inferiorly in order to avoid injury to the growth plate. The needle must also never be inserted deeper than the distance from the lateral border of the greater tubercle to the long head of biceps brachii (approximately 8 mm in the studied sample).

IO needle placement is not definitive therapy; rather, it allows for the administration of life-saving medication and fluids when intravascular access is vital. IO needles may be left in place in the marrow for up to 96 hours, but IV access is usually obtained sooner (within 6–12 hours after IO access) to reduce the risk of infection or dislodgement of the needle.^15,16^

## Conclusion

This study indicated that, based on the anatomical data collected, the humeral IO site is likely to be a safe alternative to the tibial IO site in neonates. When the IO needle is inserted as per the method discussed in this study, the risk of injury to the soft tissue and epiphyseal growth plate is minimised. Although this study was limited to a neonatal sample, we suggest that the described technique may be viable and safe in older infants and children as well.
